# Riverine food environments and food security: a case study of the Mekong River, Cambodia

**DOI:** 10.2471/BLT.22.288830

**Published:** 2022-12-08

**Authors:** Swetha Manohar, Shauna Downs, Sabina Shaikh, Sithirith Mak, Serey Sok, Elizabeth Graham, Lais Miachon, Jessica Fanzo

**Affiliations:** a Global Food Ethics Policy Program, School of Advanced International Studies, 1776 Massachusetts Avenue, Washington DC, 20007, United States of America (USA).; b Department of Health Behavior, Society and Policy, Rutgers School of Public Health, Piscataway, USA.; c Committee on Environment, Geography and Urbanization, University of Chicago, Chicago, USA.; d Department of Natural Resource Management, Royal University of Phnom Penh, Phnom Penh, Cambodia.; e Research Office, Royal University of Phnom Penh, Phnom Penh, Cambodia.; f Global Food Ethics Policy Program, Johns Hopkins School of Public Health, Washington DC, USA.

## Abstract

Rivers are critical, but often overlooked, parts of food systems. They have multiple functions that support the food security, nutrition, health and livelihoods of the communities surrounding them. However, given current unsustainable food system practices, damming and climate change, the majority of the world’s largest rivers are increasingly susceptible to environmental degradation, with negative implications for the communities that rely on them. Here we describe the dynamism and multifaceted nature of rivers as food environments (i.e. the place within food systems where people obtain their food) and their role in securing food security including improved diets and overall health. We also provide a conceptual framework that explain rivers as food environments within the broader food system and describe approaches to characterizing these food environments to better inform our understanding of how they influence food security and nutrition outcomes. Applying this framework to the Mekong River in Cambodia, we describe rivers as multifaceted wild food environments embedded within ecosystems, sociocultural and political environments and sectors of influence. We also explain the ways in which individual factors might influence how communities interact with this food environment. Developing and articulating food-related, ecosystem-specific frameworks and their constructs can guide implementation of policies aimed to improve specific public health or environmental sustainability outcomes. Our conceptual framework incorporates the multiple dimensions of rivers, which will aid future work and public health policy framing to better describe, understand and intervene to ensure protection of rivers’ biodiversity and ecosystems as well as food security, health and livelihoods.

## Résumé

Souvent négligés, les cours d'eau jouent pourtant un rôle vital dans les systèmes alimentaires. Ils remplissent de nombreuses fonctions qui contribuent à la sécurité alimentaire, à la nutrition, à la santé et aux moyens de subsistance des communautés avoisinantes. Toutefois, en raison d'un système alimentaire actuel aux pratiques non durables, de la construction de barrages et du changement climatique, la plupart des grands fleuves et rivières du monde sont de plus en plus exposés aux dégradations environnementales, avec des conséquences néfastes pour les communautés qui en dépendent. Dans le présent document, nous évoquons le dynamisme et les multiples facettes des cours d'eau en tant qu'environnements alimentaires (c'est-à-dire l'endroit où les gens se procurent de la nourriture au sein d'un système alimentaire), ainsi que la manière dont ils favorisent la sécurité alimentaire, notamment par le biais d'un régime plus équilibré et d'une meilleure santé en général. Nous fournissons également un cadre conceptuel qui définit les cours d'eau comme environnements alimentaires au sein d'un système plus vaste et décrit les approches caractérisant ces environnements, afin que nous puissions mieux comprendre l'influence qu'ils exercent sur la sécurité alimentaire et la nutrition. En appliquant ce cadre au fleuve Mékong, au Cambodge, nous dévoilons les multiples facettes de ces environnements alimentaires naturels variés qui font partie intégrante des écosystèmes, des milieux politiques et socioculturels, mais aussi des sphères d'influence. Nous expliquons en outre la façon dont certains facteurs individuels sont susceptibles d'interférer dans les interactions que les communautés entretiennent avec cet environnement alimentaire. Développer et formuler des cadres et structures liés à l'alimentation, propres à chaque écosystème, peut orienter la mise en œuvre de mesures visant à améliorer des résultats spécifiques dans le domaine de la santé publique ou de la préservation de l'environnement. Notre cadre conceptuel inclut les différents aspects des cours d'eau, ce qui aidera à mener de futurs travaux et à élaborer des politiques de santé publique pour mieux les qualifier, les appréhender et intervenir en vue d'assurer la protection de la biodiversité et des écosystèmes fluviaux ainsi que la sécurité alimentaire, la santé et les moyens de subsistance.

## Resumen

Los ríos son partes fundamentales de los sistemas alimentarios, pero con frecuencia se ignoran. Tienen múltiples funciones que favorecen la seguridad alimentaria, la nutrición, la salud y los medios de vida de las comunidades que los rodean. Sin embargo, dadas las actuales prácticas insostenibles del sistema alimentario, la construcción de embalses y el cambio climático, la mayoría de los ríos más grandes del mundo son cada vez más susceptibles a la degradación medioambiental, con implicaciones negativas para las comunidades que dependen de ellos. En este documento, se describe el dinamismo y la naturaleza polifacética de los ríos como entornos alimentarios (es decir, el lugar dentro de los sistemas alimentarios donde las personas obtienen sus alimentos) y su función para garantizar la seguridad alimentaria, incluida la mejora de los hábitos alimentarios y la salud en general. Asimismo, se proporciona un marco conceptual que explica los ríos como entornos alimentarios dentro del sistema alimentario en su conjunto y se describen enfoques para caracterizar estos entornos alimentarios con el fin de comprender mejor cómo influyen en los resultados de la seguridad alimentaria y la nutrición. Mediante la aplicación de este marco al río Mekong en Camboya, se describen los ríos como entornos alimentarios silvestres multifacéticos integrados en ecosistemas, entornos socioculturales y políticos y sectores de influencia. Además, se explican cómo los factores individuales pueden influir en la interacción de las comunidades con este entorno alimentario. El desarrollo y la articulación de marcos específicos de los ecosistemas relacionados con la alimentación y sus constructos pueden orientar la aplicación de políticas destinadas a mejorar determinados resultados en materia de salud pública o sostenibilidad medioambiental. El marco conceptual que se presenta incorpora las múltiples dimensiones de los ríos, lo que ayudará en futuros trabajos y en la formulación de políticas de salud pública a describir, comprender e intervenir mejor para asegurar la protección de la biodiversidad y los ecosistemas fluviales, así como la seguridad alimentaria, la salud y los medios de subsistencia.

## ملخص

تمثل الأنهار أجزاءً مهمة من النظم الغذائية، ولكن غالبًا ما يتم تجاهلها. فيوجد لديها وظائف متعددة تدعم الأمن الغذائي، والتغذية، والصحة، وسبل العيش في المجتمعات المحيطة بها. إلا أنه نظرًا لممارسات النظام الغذائي غير المستدامة الحالية، وبناء السدود، وتغير المناخ، فإن غالبية أكبر أنهار العالم معرضة بشكل متزايد للتدهور البيئي، مع ما يترتب على ذلك من آثار سلبية على المجتمعات التي تعتمد عليها. نحن هنا نصف الديناميكية والطبيعة المتعددة الأوجه للأنهار كبيئات غذائية (أي المكان الموجود في النظم الغذائية حيث يحصل الناس منه على طعامهم)، ودورها في تأمين الأمن الغذائي بما في ذلك النظم الغذائية المحسنة، والصحة العامة. نحن نقدم أيضًا إطار عمل مفاهيمي يشرح الأنهار على أنها بيئات غذائية ضمن نظام غذائي أوسع، ويشرح أساليب لتوصيف هذه البيئات الغذائية لتوعية فهمنا بشكل أفضل لكيفية تأثيرها على الأمن الغذائي ونتائج التغذية. بتطبيق إطار العمل هذا على نهر ميكونج في كمبوديا، نحن نصف الأنهار على أنها بيئات غذائية برية متعددة الأوجه، مدمجة في النظم البيئية، والبيئات الاجتماعية والثقافية والسياسية، وقطاعات التأثير. نحن نوضح أيضًا الطرق التي قد تؤثر بها العوامل الفردية على كيفية تفاعل المجتمعات مع هذه البيئة الغذائية. يمكن أن يؤدي تطوير وتوضيح الأطر المتعلقة بالأغذية والخاصة بالنظم الإيكولوجية وبنياتها إلى توجيه تنفيذ السياسات الهادفة إلى تحسين نتائج محددة تتعلق بالصحة العامة أو الاستدامة البيئية. يشتمل إطار العمل المفاهيمي لدينا على الأبعاد المتعددة للأنهار، والتي سوف تساعد العمل المستقبلي وتأطير سياسات الصحة العامة من أجل وصف وفهم وتدخل أفضل، لضمان حماية التنوع البيولوجي والنظم البيئية للأنهار، وكذلك الأمن الغذائي، والصحة، وسبل العيش.

## 摘要

河流是食物系统的重要组成部分，但往往被人们忽视。它们具有维持周围社区的食物安全、营养、健康和生计的多种功能。然而，鉴于目前不可持续的食物系统实践、筑坝和气候变化，世界上大多数大河越来越容易受到环境退化的影响，对依赖这些河流的社区产生了负面的影响。在这里，我们介绍了河流作为食物环境（即食物系统中人们获取食物的地方）的活力和多方面性，以及它们在确保食物安全（包括改善人们饮食和总体健康）方面所起的作用。我们还提供了一个概念框架，解释了河流在更广泛的食物系统中作为食物环境的作用，并介绍了描述这些食物环境的方法，以更好地了解它们如何影响食物安全和营养状况。将此框架应用于柬埔寨的湄公河流域，我们将河流定义为嵌入生态系统、社会文化和政治环境及其所影响部门的多面性野生食物环境。我们还解释了个别因素可能如何影响社区与食物环境相互作用的方式。制定和阐明与食物相关的特定生态系统框架及其结构，可以指导旨在改善特定公共卫生或环境可持续发展状况的实践。我们的概念框架纳入了河流的多个维度，这将有助于未来开展工作和制定公共卫生政策的框架，以便更好地描述、理解和实施干预，确保河流的生物多样性和生态系统以及食物安全、人类的健康和生计得到保护。

## Резюме

Реки являются важнейшей, но часто упускаемой из виду частью продовольственных систем. Они выполняют множество функций, способствующих обеспечению продовольственной безопасности, питания, здоровья и средств к существованию окружающих их сообществ. Однако в условиях существующих неэкологичных практик в продовольственной системе, зарегулирования стока плотинами и изменения климата экологическое состояние большинства крупнейших рек мира постоянно ухудшается, что негативно сказывается на зависящих от них сообществах. В этой статье описывается динамизм и многогранность рек как продовольственной среды (то есть как места в продовольственных системах, откуда люди получают пищу) и их роль в обеспечении продовольственной безопасности, включая улучшение рациона питания и общего состояния здоровья. Также представлена концептуальная схема, в которой реки рассматриваются как продовольственная среда в рамках более широкой продовольственной системы, и описаны подходы к определению характеристик этой продовольственной среды для лучшего понимания ее влияния на продовольственную безопасность и результаты в сфере питания. Применяя эту схему к реке Меконг в Камбодже, авторы описывают реки как многогранную среду обитания диких продовольственных ресурсов, встроенную в экосистемы, социокультурную и политическую среду и секторы влияния. Авторы также объясняют, каким образом отдельные факторы могут влиять на то, как сообщества взаимодействуют с этой продовольственной средой. Разработка и разъяснение схем и концепций, связанных с продуктами питания в конкретных экосистемах, могут определять реализацию политики, направленной на улучшение конкретных результатов в области общественного здравоохранения или экологической устойчивости. Разработанная концептуальная схема включает в себя многочисленные показатели состояния рек, что поможет в ходе дальнейшей работы и разработки политики в области общественного здравоохранения добиться лучшего описания, понимания и вмешательства в целях обеспечения защиты биоразнообразия и экосистем рек, а также обеспечения продовольственной безопасности, здоровья и средств к существованию.

## Introduction

Climate change affects food security and the nutritional well-being of populations worldwide. By 2050, an estimated 183 million additional people will be at risk of hunger linked directly to climate change.^1^ In turn, current food systems, responsible for a third of total global greenhouse gas emissions, hasten climate change and degradation of ecosystems leading to significant deforestation and biodiversity loss.^2^ Ecosystems are crucial components of our food system and serve as food environments, that is the interface at which consumers interact with the broader food system by making choices within a physical, economic, political and sociocultural context.^3^

River ecosystems have historically provided numerous food-related benefits to humans including irrigation, livelihoods and as a vessel for food. Rivers can be described as nutrient highways across the earth’s surface, transporting sediment and water, sequestering carbon from the atmosphere, and connecting and storing immense biodiversity through aquatic life. The flow and transportation of sediment create environments for cultivation (e.g. rice farming), with river deltas being one of the world’s most agriculturally productive areas.^4^ Rivers support approximately a third of all global food production, and an estimated 70% of freshwater from rivers is used for agriculture.^5^^,^^6^ In addition, in countries where consumption data is available, freshwater fish, primarily from inland fisheries, are estimated to be the primary animal source protein consumed by more than 119 million people.^7^ Rivers are therefore integral to food systems and a critical resource for assuring nutritional well-being and food security. Their multidimensional nature gives them a unique position as a natural, wild food environment, a concept not well-characterized in the literature or in the public health policy sphere.

Emerging research focuses on the importance of food environments as a point of convergence between food, human beings, choice and acquisition.^8^^,^^9^ Environmental, economic, and other shocks can affect food environments, which are dynamic spaces embedded in interdependent global food systems. Characterizations of food environments have primarily focused on the built food environment, such as supermarkets, corner stores and kiosks. The literature remains scant on depictions of informal and wild food environments (e.g. forests, rivers) as a source of food security despite the importance of these environments and communities’ reliance on them, especially in low- and middle-income countries.^10^

Livelihood and income-generating activities related to rivers are equally dynamic and include fishing, farming and trade. However, given the unsustainability of modern food systems and human activities coupled with climate change, 30 out of 47 of the world’s largest rivers are under threat of human water insecurity and biodiversity loss ultimately linked to food productivity loss.^11^ These unsustainable food systems have implications for food security, nutrition and health for river-dependent populations. While there is broad recognition of these connections, the role of rivers as a crucial food environment in protecting against food insecurity and malnutrition is rarely described. This evidence gap can be conceivably attributed to the imbalance of research undertaken in high- versus low-resourced settings where urbanization and market-driven economies have given rise to primarily built food environments versus a more heterogenous food environment typology.^8^^,^^12^

This paper will expand on the dynamism and multifaceted nature of rivers as food environments and their role in securing food security. By using the Mekong River in Cambodia as a case study, we will discuss how to understand rivers as food environments, how rivers underpin food security and their unique features and threats. Further, we offer two conceptual frameworks. The first allows for the conceptualization of rivers as a food environment, while the second establishes a structure to assess rivers as food environments and highlights existing assessments that can be applied. Both frameworks can be useful constructs in understanding how to develop and implement policies related to improving food security, diets and public health outcomes.

## Rivers as a food environment

**Fig. 1:**
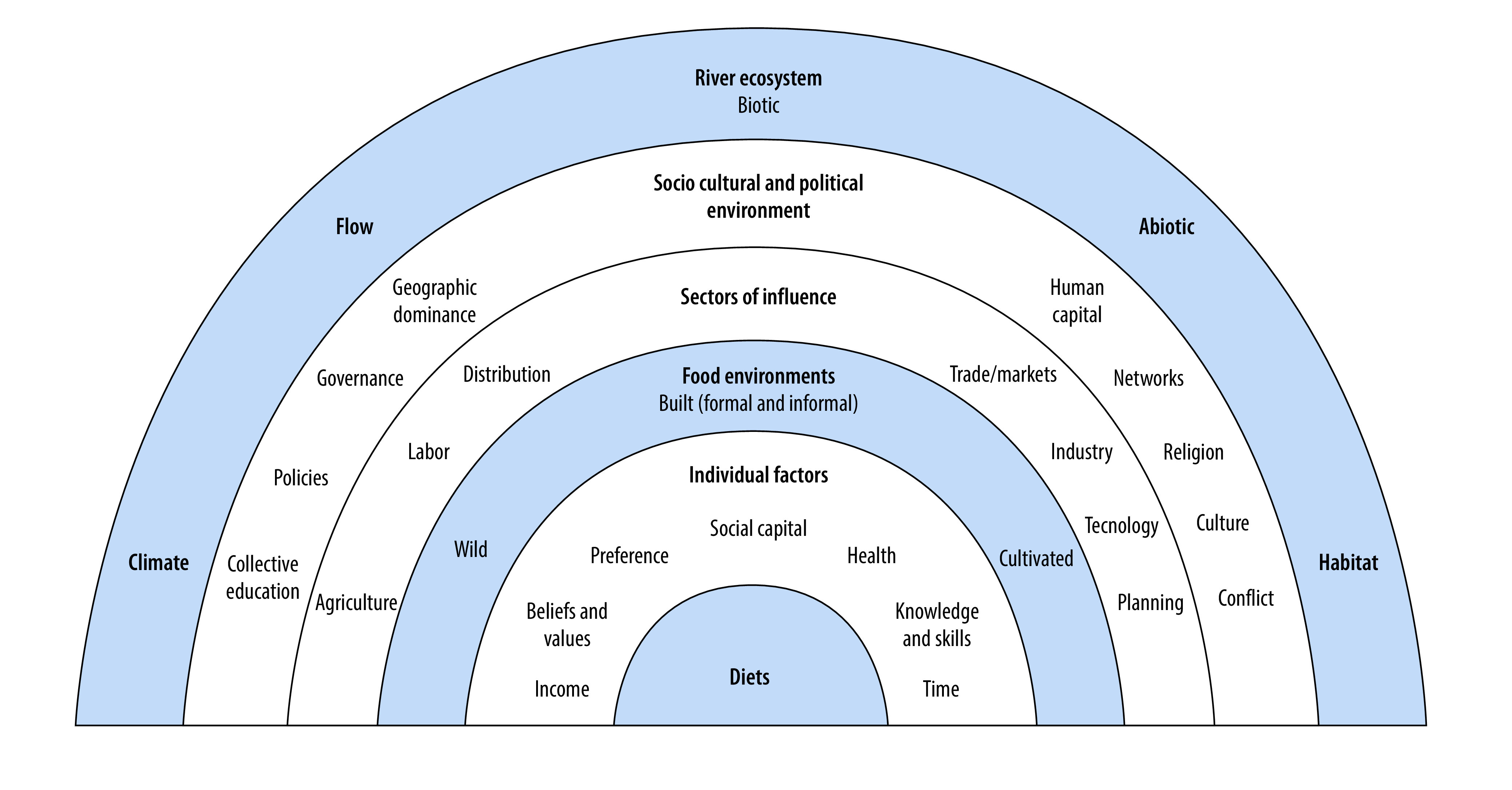
Conceptual framework for riverine food environments

Note: We adapted a schematic socioecological model of food environments^13^ to reflect riverine food environments.

We adapted a schematic socioecological model of food environments^13^ to position rivers as food environments within the broader food system and to reveal its broader ecosystem (Fig. 1). We selected this conceptual framework based on its grounding in the socioecological model and theory, in which most of the food environment literature is rooted.^14^ The framework emphasizes the multilevel linkages that ultimately impact individual-level dietary intake and nutrition, bringing to focus the interrelated nature between environments and people. Furthermore, this evidence-based framework situates its conceptual thinking in low- and middle-income country contexts and prominently features the wild food environment in addition to the built food environment. The framework advances our understanding by demonstrating how rivers are pivotal food environments through which food security and healthy diets are secured while also being shaped by and interacting with other factors. We have adapted the framework to reflect the entire river ecosystem and its constant interaction with the sociocultural and political environment, various sectors of influence, food environments and ultimately individual-level factors and diets. These interactions can be bidirectional, whereby shifts in sectors of influence such as river-based livelihoods or unregulated market activity can shape food environments as much as consumer food preferences that are river-reliant can drive changes in river ecosystems.

We first considered the environmental and political aspects of rivers and food environment attributes more broadly when adapting the original framework. Next, we considered how the unique attributes of rivers lend themselves to function as food environments. Table 1 expands the conceptual framework by describing how factors included in each band in the model relate to riverine food environments. We generated Table 1 through an iterative approach where we assessed each factor for its applicability to riverine food environments using peer-reviewed literature and relying on the authors’ collective interdisciplinary expertise. The river ecosystem band (Fig. 1) is informed by river ecology literature as it relates to food security.^15^^,^^16^ The literature search was conducted with variations of the following terms: “wild food environments,” “natural food environment,” “river,” “food environment,” “food security,” “Mekong River,” “Cambodia,” “food environment,” “sustainable,” “river food ecology,” “low middle income countries.” We specifically looked for studies that discuss rivers as food environments or discuss wild food environments in riparian populations, as well as literature on the dependence on the Mekong River (and other rivers) as a direct source of food in low- and middle-income countries. While the table is not exhaustive, its relevance is supported by the evidence reviewed to help expound factors captured and depicted in the conceptual framework in a way that is illustrative.^5^^,^^10^^,^^13^^,^^17^^–^^19^ The identification, organization and assessment of this information serve to bolster research and policy considerations on river systems as unique food environments.

**Table 1:** Conceptual description of factors outlined in the conceptual frameworks

Layer, factor^a^	Relevance of factors to rivers as food environments
**River ecosystem**	
Biotic	Living plants (algae) and creatures (fish, frogs, crocodiles, shrimp, dolphins, geckos), soil, sediment
Abiotic	Chemical and physical elements such as water, dirt, rocks, sunlight, oxygen, temperature, sand, pH
Climate	Droughts, floods, climate change exacerbations (and detractions) of extreme events, water temperature change, cyclical seasonal patterns
Flow	Flow paths, watershed hydrology, river connectivity, flood pulse ecosystem
Habitat	Riparian zone, biodiverse ecological communities, human settlements
**Sociocultural and political environment**	
Collective education	Non-traditional knowledge, ecosystem-related traditional indigenous knowledge, conservation practices
Policies	Energy (hydropower), conservation, environmental, water, economic, microfinance, agriculture and fishery, resettlement
Governance	Transboundary cooperation, resource management, Mekong River Commission
Geographical dominance	GDP, economic power, political power
Human capital	Employment in fisheries, aquaculture, and all river-related activity (trade, processing, boat builders and maintenance, etc.), within country migration away from rural areas (to cities for factory and construction work)
Networks	Intergovernmental organizations (Mekong River Commission), conservation groups, community fishery groups, worker groups and unions, agriculture cooperatives, rural-urban migration networks
Religion	Sacred areas, ritual space and practices (e.g. Hinduism, Buddhism, Animism)
Culture	Folk narratives, river-related place-based identities, principles, norms and values, indigenous meaning-making related to rivers, origins of civilizations (Khmer civilizations of 800 AD), recreation
Conflict	Drug trade, land ownership, illegal fishing, damming, water access rights
**Sectors of influence**	
Agriculture	Fisheries and aquaculture, staple crop (rice), irrigation
Labour	Labour migration away from rural fishing-related economies or river-based livelihoods
Distribution	River transportation of commodities including food, medicine
Trade/markets	River trade of goods and services, barter of river-related products
Industry	Construction, milling and/or processing, hydroelectric power, food processing, food safety
Technology	Satellite imagery for river planning, agriculture and aquaculture technology
Planning	Urban planning, river basin planning
**Food environments**	
Wild	River, such as fish, river plants and other animals
Built (formal)	Riverside restaurants and kiosks
Built (informal)	Kiosks and food stalls along riverbanks, floating markets, river vendors
Cultivated	Horticulture, such as home gardens, rice paddies, fisheries, aquaculture, irrigation for cultivation
**Individual factors**	
Income	Employment in agriculture, fisheries, aquaculture, forestry and other river-based jobs and livelihoods
Beliefs and values	Intergenerational river communities, sense of place and identity with land, indigenous value systems related to rivers, water purity
Preferences	Personal taste and preferences for aquatic plants and animals
Social capital	Place-based social networks that allow reciprocity and exchanges
Health	Human health, access to clean water and sanitation, morbidities
Knowledge and skills	Formal and informal training, skills for using and/or managing river systems and resources, culinary skills
Time	Time allocation, restrictions and burdens
**Diets **	Contribution of river plants and creatures to diets and nutrition, macro- and micronutrient content of river plants and creatures

GDP: gross domestic product.

^a^ Layers are illustrated in Fig. 1 and listed from distal to proximate.

Food environments can be characterized as built or natural, the latter comprising both cultivated and wild food environments. Cultivated natural food environments make a significant contribution to the diets of subsistence farmers and rural communities through the production of staple crops, supplemental gardens that produce fruits and vegetables, and the rearing of livestock and aquaculture, and utilization of their by-products, such as eggs and milk. Cultivated food environments are often dependent and intrinsically linked with wild natural food environments, which include forests, jungles, rivers and lakes. These environments are particularly important for increasing access to nutrient-rich foods, including animal source foods, leafy greens and other vegetables and fruits,^19^ and can increase resilience of households to shocks.^20^^,^^21^ Rivers act as wild food environments from which food (fish and edible aquatic plants) is procured,^22^ but also as cultivated food environments where staple crops are grown. The riverbanks can serve as informal built food environments (habitual wet markets and mobile fish vendors) as well as formal built food environments where restaurants are situated. But river bodies also serve as a space for selling foods and allow for trade and transportation of food. As such, they cut across the different food environment types and the food system itself. From a public health policy standpoint, understanding the dynamic role of rivers as food environments is critically important in determining where, when and how to act to not only protect food security and diets, but to instil resilience of the rivers themselves as important food sources.

## The case study

The Mekong River traverses south-east Asia and is shared by six countries – Cambodia, China, Lao People's Democratic Republic, Myanmar, Thailand and Viet Nam – where approximately 65 million people live in its basin.^23^ In Cambodia, the river splits the country, flowing over 500 km, and comprises 39 river basins. Nearly 80% of the country’s 16.7 million people rely on the basin for their livelihoods and other resources.^23^ The Mekong River connects with the Tonle Sap Lake which, with its unique flood pulse hydrology, functions as the largest inland fishery in the world.^17^ Currently, Cambodia’s food systems are transitioning – they are expanding and transforming, moving away from smallholder self-sufficiency agriculture to more significant commercial production. However, with 75% (12.7 million) of the Cambodian population still living in rural areas, the Mekong River and its tributaries remain crucial to ensuring the country’s food security.

The downstream impacts of hydropower dam construction on the Mekong River include altered water and sediment flow to the delta.^24^ The alterations to the biophysical and hydrological system have significant effects on agricultural and fishery production, particularly for small-scale operators who are already constrained by the lack of resources and political agency, and threaten their livelihoods and agricultural ways of life.^24^

The Mekong River Commission, established in 1995, provides a framework to promote cooperation in the region, including through data collection to facilitate river management.^25^ However, the commission is limited in its capacity due to the lack of membership of key upstream countries, including China, and the mode of cooperation is not legally binding. Thus, governments may ignore the commission’s recommendations if they impede governmental plans. For example, not all Mekong River Commissions’ member states have been transparent about dam development.^26^ Further, other regional cooperation agreements, such as the frameworks of the Mekong-Lancang Cooperation and Mekong-Republic of Korea Cooperation, are at times at odds with the commission and undermine its efforts.^25^

### Applying the framework

We expand our evaluation of the Mekong River as a food environment, starting at the band of food environments itself (Fig. 1), and discuss its interaction with individual factors and the diets of the Cambodian population. As described earlier, the river functions as a wild, cultivated and informal built food environment. Total fisheries production is estimated at 9.5 million metric tonnes of fish in 2018 which include capture fisheries and aquaculture products.^27^ These yields support an average annual consumption of approximately 63 kg/capita in Cambodia in contrast to 20 kg/capita worldwide.^28^ The distinctive flood pulse system of the Mekong River and Tonle Sap Lake is responsible for production of three quarters of the country’s dominant agricultural product, rice.^29^

Wild foods frequently harvested for consumption include snails, frogs, prawns, crabs, insects, waterbirds and aquatic plants.^30^ The proportion to which river wild foods contribute to the overall caloric and nutrient intake of the Cambodian population is not well characterized, however, an estimated >90% of household catch of aquatic plants and animals (besides fish) is consumed by household members.^30^ Beyond cultivation, the sale of food harvested from the river along with other types of food items (including processed foods) are sold on and around the river in both informal and formal markets.

Individual factors that interact with both the river as a food environment and dietary intake include values and beliefs, income, social capital, health, knowledge, skills and time. Cultural practices and celebrations, including the Water Festival and other religious customs, reflect belief and value systems at the individual and broader community level.

The reliance of the rural Cambodian population on the Mekong River for their livelihood and food security is high: approximately 80% of river communities directly rely on the river and lake for food and livelihoods and 45% of its households undertake fishing-related income activities.^31^ Increasingly, Tonle Sap Lake communities have experienced reductions in catch quantity due to overfishing and ecological threats, but have limited access to resources for adapting to the situation.^32^ Opportunities exist to assess the sustainability attributes of riverine food environments that reflect adaptive practices to climate and livelihood changes. Other individual factors include social capital; the links between social capital and food security are well-established,^33^ but there is limited evidence in the Cambodian context. Of note, low accessibility to both social and human assets among lower Mekong River communities has impeded livelihood strategies, which has resulted in social incoherence.^34^ Globally, maternal education is positively associated with both the mother’s own and her child’s dietary intake, a relationship that can be modified with food availability.^35^ Time allocation, especially women’s time and restrictions placed on their time, have been associated with poor child feeding practices and the mother’s own quality of diet.^36^ In recent decades, the dramatic increase in migration of women from rural areas to cities has affected both women’s and children’s diets. Ultimately, these individual factors can shape Cambodian diets, which are usually comprised of rice and fish as a primary protein source, both of which are river dependent. White fish (*trey riel*) commonly used to make fish paste (*prahok*) creates an affordable animal protein with a longer shelf life. These interactions of individual factors with riverine food environments to support food acquisition are important in a context where the burden of malnutrition and food insecurity is high.^37^ For example, in 2021, during the coronavirus disease 2019 pandemic, estimates of household food security revealed that 32% of households experienced moderate and/or severe food insecurity, with the poorest households carrying a disproportionate burden. Among the poorest households, an estimated 55% experienced such food insecurity.^38^

When considering the more distal layers of the conceptual framework, the literature shows evidence of different contextual factors in Cambodia influencing the Mekong River and its tributaries as a food environment. Sectors of influence, given their relationship with the Mekong River, are the agricultural, labour, markets, distribution, industry and technology sectors in Cambodia. Rice farming and fisheries livelihoods have been critical contributors to gross domestic product growth since the 1990s.^39^

The sociocultural and, especially, the political environment of the Mekong River, is complex. There are currently an estimated 132 hydropower dams built, under construction or planned on the tributaries of the lower Mekong River.^40^ While electrification is critical for Mekong River populations, there are questions of how to reconcile electrification projects with trade-offs of sustainable development, displacement and other risks to populations living downstream of the dams. The construction of upstream dams in both China and Lao People's Democratic Republic has directly affected water levels by restricting water flow, removing the anticipated seasonality of the rise and fall of water levels and blocking fish from upstream movement and sediment flow.^24^^,^^41^ Projected production loss alone on capture fisheries in Cambodia is between 40% and 57% by 2030.^28^ This loss would imply anywhere between 6.4 million and 21.1 million people losing their main protein source.^28^ Without reliable river flows, seasonal food insecurity and a decline in agricultural productivity can affect an estimated 70% of inhabitants of river communities that rely on the river for fisheries livelihoods and food cultivation.^40^ Conflict over water rights, illegal fishing practices and inconsistent regional cooperation are the major challenges in the transboundary Mekong River basin governance.^42^

Finally, the river ecosystem comprises factors that reflect biotic, abiotic, climate, flow and habitat features of this ecosystem. Climate change is altering the Mekong ecosystem in various ways including its hydro-ecological conditions, sediment and nutrient flux, and vegetation growth^43^^,^^44^ as well as the ability to harvest and grow food in and around the Mekong River.^45^ Extreme droughts and floods have devastating consequences on the ecology of the river with future scenarios projecting increased risks of both.^46^^,^^47^ Human activities in and along the river have affected flow dynamics, while significant increases in populations living in the lower Mekong area has put pressure on natural resources in the region. A shift has begun, moving away from smallholder farming towards more industrialized, specialized agriculture and cash crop systems, and farmers moving towards off-farm livelihoods.^48^ Geophysical and anthropogenic forces have altered the agro-ecological landscapes of the basin, which has seen increasing deforestation due to forest-cover change and land-use conversion from forests to farmland.^49^ Unsustainable agricultural practices have also been linked to dramatic habitat loss over the past 10 years (2300 km^2^ of seasonally flooded habitat was lost to agriculture, with another 400 km^2^ recently burnt), almost twice what was predicted (1300 km^2^) to occur between 2010–2040.^50^ In addition, Cambodia faces the highest forest loss and deforestation rates among all countries in the Mekong basin, due to illegal logging, with significant negative impacts on floodplain productivity.^49^

## Assessing river food environments

**Fig. 2:**
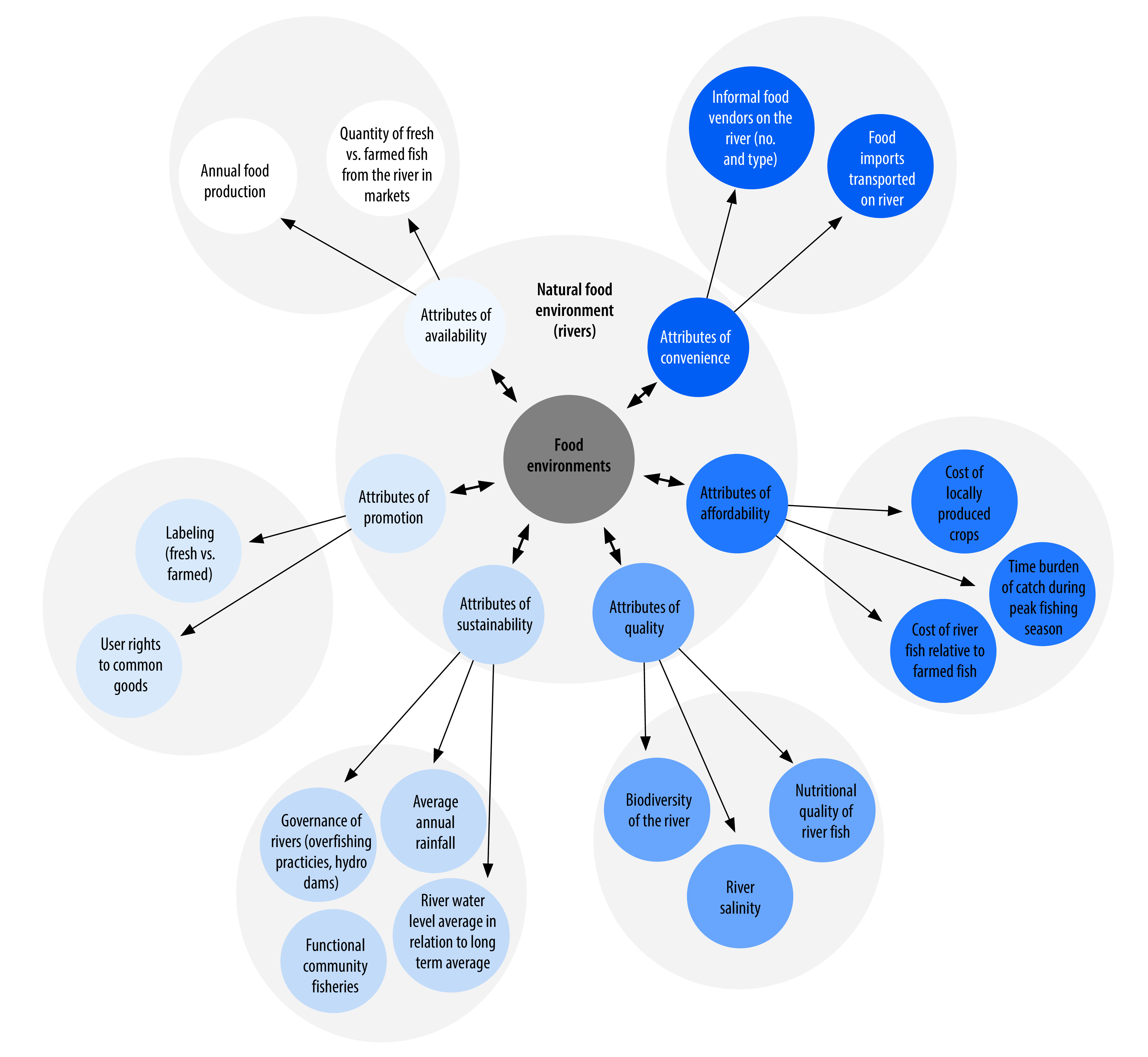
Key elements of multidimensional riverine food environments

Policy-makers need new tools to understand how riverine food environments are changing, the impacts on food security and diets, and how and where to intervene in the context of climate change. The framework in Fig. 2 illustrates the types of interdisciplinary assessments that exist to evaluate riverine food environments.^10^^,^^13^ We highlight the six main attributes of food environments typically used for food environment assessment: (i) availability; (ii) affordability; (iii) convenience; (iv) promotion; (v) quality; and (vi) sustainability, and illustrate the corresponding measurements.^3^^,^^13^^,^^21^^,^^51^ While tools may exist to assess certain (not all) attributes, these assessments have not been conducted in a holistic manner for riverine food environments.^13^ This lack of assessment may relate to the complexity and breadth of such assessments. Given the uniqueness of riverine food environments, their assessment requires a multitude of tools from various disciplines not limited to ecology, hydrology, economics, food science, public health and nutrition. These attributes are influenced by macrolevel factors (Fig. 1) and river ecosystems themselves that respond to climatic change and other stressors. Understanding the availability, quality, affordability, convenience, promotion, and sustainability attributes of food environments (Fig. 2) can help public health policy-makers and practitioners in developing, prioritizing and implementing localized policies. Such policies can improve those attributes to promote healthy and sustainable dietary choices and options for populations. For example, the attributes of quality shows how biodiversity is crucial for rivers to be fully functional food environments. Public health policy-makers can inform and collaborate with agriculture and fisheries ministries to ensure biodiversity of rivers; this protection and promotion of biodiversity can positively affect health. The dynamic nature of these riverine food environments adds an additional challenge in that they are kinetic, they change markedly based on the season and they are heavily influenced by climate variability and change.^13^^,^^19^^,^^42^ This variability makes measuring aspects of sustainability and temporal features of these environments even more critical.

Fig. 1 and Fig. 2 recognize the relationships, effects and trade-offs across the full riverine food environment system, and all the domains and dimensions of those relationships. Without a clear framing of those relationships and a conceptual understanding of where to intervene, it is challenging to account for the system dynamics and complexities and act across a range of policy interventions. Despite the existence of many food system frameworks for policy-makers, very few have incorporated ecosystems into them, and even fewer incorporate the role of rivers, which are crucial ecosystems for food security.^31^ The application of the frameworks presented here allows for an approach to monitoring rivers and advocating their protection, management and utilization with greater purpose for food security and nutritional well-being. Further policy implications include paying greater attention to the downstream effects of energy planning including the pathways by which dam construction and land use decisions alter hydrology and drive food insecurity across borders and jurisdictions.

## Conclusion

If we are to understand how to produce, acquire and consume food within planetary boundaries to support nutritional well-being, we cannot do so without characterizing and protecting ecosystems-based food environments that serve as a critical interface for food acquisition within larger systems. Rivers contribute in multiple ways to food security, diets and nutrition outcomes. In some areas of the world, such as the Mekong River basin, rivers are critical for food security and the livelihoods of millions of people.

While rivers have been deeply studied, we know much less about rivers when framed as dynamic food environments. What we do know is that key attributes of rivers provide critical contributions to multiple sustainable development outcomes. When framing rivers as food environments, assessments need to be multilevel, multisectoral and interdisciplinary to adequately describe, understand and identify points of policy and programmatic interventions. This framing is to ensure that biodiversity and ecosystems of rivers are protected, as well as to improve human food security and livelihoods. To undertake such assessments, more tailored information, approaches and tools are needed to assess their dynamic nature. The assessment should include not only their contributions to healthy diets, but to environmental sustainability and resilience and the overall management of this critical natural resource relied upon by communities living near or in these environments. Further, articulating the pathways by which economic, social and environmental policy interventions affect riverine food environments is important for assessment refinement and for course corrections to ensure trade-offs that create adverse impacts on rivers and riverine communities are minimized.
